# Assessing the Impact of Insecticide Resistance on Vector Competence: A Review

**DOI:** 10.3390/insects13040377

**Published:** 2022-04-12

**Authors:** Alan E. Juache-Villagrana, Victoria Pando-Robles, Selene M. Garcia-Luna, Gustavo Ponce-Garcia, Ildefonso Fernandez-Salas, Beatriz Lopez-Monroy, Iram P. Rodriguez-Sanchez, Adriana E. Flores

**Affiliations:** 1 Facultad de Ciencias Biologicas, Universidad Autonoma de Nuevo Leon, Av. Universidad s/n Cd. Universitaria, San Nicolas de los Garza 66455, Nuevo Leon, Mexico; juva2030083@uanl.edu.mx (A.E.J.-V.); selene.garcialn@uanl.edu.mx (S.M.G.-L.); gustavo.poncegc@uanl.edu.mx (G.P.-G.); ildefonso.fernandezsl@uanl.edu.mx (I.F.-S.); beatriz.lopezmr@uanl.edu.mx (B.L.-M.); iram.rodriguezsa@uanl.edu.mx (I.P.R.-S.); 2 Centro de Investigacion Sobre Enfermedades Infecciosas, Instituto Nacional de Salud Publica, Cuernavaca 62100, Morelos, Mexico; victoria.pando@insp.mx

**Keywords:** insecticide resistance, insecticide exposure, vector competence, pathogen transmission

## Abstract

**Simple Summary:**

Insects transmit a wide variety of pathogens, including parasites, bacteria, and viruses, to human and economically important crops. Since pathogen transmission threatens public health and economic activities, insecticides are the main strategy to control insect populations. The continued use of insecticides has led to resistant populations where chemicals are no longer effective. It is unknown if insecticide resistance (IR) could impact insects’ other characteristics, such as their ability to infect, maintain infection, or transmit pathogens, a trait known as vector competence (VC). In this review, we analyze the literature that involves the study of VC and IR or insecticide exposure in three main approaches; studies conducted in the field versus laboratory-designed experiments, the impact of insecticide exposure on pathogen transmission, and studies performed on vectors of crop pathogens. The evidence points out three different patterns where enhancement, impairment, or neutral effects are seen between IR and VC. It is of great concern that IR could enhance VC since it increases the risk of epidemics. More detailed and standardized studies are needed to confirm this relationship. Finally, results from this investigation could help create evidence-based vector control programs.

**Abstract:**

The primary strategy to avoid adverse impacts from insect-mediated pathogen transmission is the chemical control of vector populations through insecticides; its continued use has led to insecticide resistance and unknown consequences on vector competence. This review aims to systematically analyze and synthesize the research on the influence of insecticide resistance (IR) on vector competence (VC). Thirty studies met the inclusion criteria. Twenty studies, conducted either in laboratory or field settings, described the influence of phenotypic insecticide resistance and mechanisms on VC in vectors of human pathogens. Seven studies showed the effect of exposure to insecticides on VC in vectors of human pathogens. Three studies reported the influence of phenotypic resistance and mechanisms on VC in crop pests. The evidence shows that IR could enhance, impair, or have no direct effect on VC in either field or laboratory-designed studies. Similar positive and negative trends are found in pest vectors in crops and studies of insecticide exposure and VC. Even though there is evidence that exposure to insecticides and IR can enhance VC, thus increasing the risk of pathogen transmission, more investigations are needed to confirm the observed patterns and what implications these factors could have in vector control programs.

## 1. Introduction

Insects have a close relationship with humans. They participate in activities that benefit human well-being (i.e., pollination) and exert adverse effects such as those observed in public health, crops, hygiene, and other sectors [1]. On the side of unfavorable impacts, we can highlight the insects’ role as vectors of diseases. For example, mosquitoes (i.e., Culicidae), triatomine bugs (Reduviidae), blackflies (Simuliidae), and lice (Pediculidae) affect human health by transmitting arboviruses, parasites, or bacteria [2]. Additionally, aphids, whiteflies, and thrips transmit pathogens to economically important crops [3]. Together, vectors of human and crop pathogens cause considerable economic losses due to human health costs and lower agricultural production [4].

This capacity of insects to transmit pathogens is known as vector competence (VC). This trait defines the intrinsic capacity of an organism to acquire, maintain replication, disseminate, and transmit a pathogen. VC is a complex trait influenced by factors such as the genetic background of hosts and insects, strain and genotype of pathogens, and other aspects associated with environmental variables such as temperature [5,6]. For example, many studies have evaluated the VC of *Aedes aegypti*. It has been determined that the extrinsic incubation period (the time needed for a mosquito to become infectious) is shorter at higher temperatures [7]. Additionally, bacterial symbionts affect VC by shaping immune responses [8]. Furthermore, Souza-Neto et al. [9], in a systematic review of VC literature in different populations of *Ae. aegypti* did not find any record of a fully refractory natural population to virus infection; however, there are populations completely susceptible to Zika, dengue, and chikungunya viruses, demonstrating differential regulation of VC. 

Studies of VC are heterogeneous (e.g., pathogen challenges performed through intrathoracic injections vs. membrane feeding), and experimental limitations are present (e.g., lack of model animals that mimic human pathogenesis). There are several reports giving a detailed description of past and current knowledge of VC in mosquitoes [9,10,11,12,13,14].

The primary strategy to avoid adverse impacts of insects on public health or agriculture is the use of pesticides. Pesticides are molecules used to destroy, prevent, or repel insects that are a nuisance to humans [15]. Nowadays, various chemicals are applied to control insect populations; according to their structure and synthesis, the diversity of insecticides includes chlorinated hydrocarbon compounds, organophosphates, carbamates, pyrethroids, neonicotinoids, formamidines, and other molecules, plus botanical and microbial agents [16]. The continued use of insecticides has had unintended consequences, particularly the emergence of resistant populations in both human and crop insect vectors. Given many reports on the ineffectiveness of chemical agents in controlling insect populations, we can conceptualize insecticide resistance (IR). This phenomenon is defined as the decrease in the susceptibility of an insect population to a previously effective insecticide caused by its continued use and/or possible cross-selection with other chemical substances, which arises through genetic, physiological, or behavioral changes and is also a hereditary trait [17,18,19].

IR has been described in vectors regarding a broad spectrum of chemical compounds, including organophosphates [20,21,22], among others. Four mechanisms have been determined to reduce the efficacy of pesticides: changes in insect behavior, thickening of the insect cuticle, increased activity of detoxifying enzymes, and modification of the target site [23,24,25]. The more studied IR mechanisms are target site modification and detoxifying enzyme alteration. For example, there is a vast amount of literature concerning mutations in the voltage-gated sodium channel (VGSC), known together as knockdown resistant mutations (kdr mutations, hereafter), as well as mutations in the acetylcholinesterase gene (referred to as Ace mutations hereafter). These mutations are related to pyrethroid and DDT resistance and organophosphate and carbamate resistance, respectively [26,27]. On the other hand, different enzymes participate in detoxification events leading to metabolic resistance. For instance, mixed-function oxidases are greatly involved in pyrethroid resistance [28,29], along with glutathione S transferases and esterases [30,31]. It has been shown that IR affects current efforts in vector control to prevent the emergence of epidemics of emerging or re-emerging diseases such as chikungunya [32,33]. The same has been observed in pest vectors of important crops such as soybeans and tomatoes [34,35,36].

On the other hand, little is known about the impact of IR on VC. Given the actual scenario in which IR affects vector control, we aimed to systematically synthesize and analyze the research on the effect of IR on VC in vector species that impact human health or crops. It is important to note that we only reviewed research that describes experimental procedures that directly link IR and VC. We divided this systematic review into three sections. The first analyzes research findings in the field or laboratory-designed experiments. The second focuses on the effect of exposure to insecticides on the transmission of pathogens, and the last focuses on the vector research conducted on crop pests.

## 2. Materials and Methods

### 2.1. Search Strategy and Eligibility Criteria

We used the guidelines described by PRISMA-P (Preferred Reporting Items for Systematic Review and Meta-Analysis Protocols) for this systematic review. PubMed, Google Scholar, Science Direct, and CONRICYT databases were searched using a combination of two keywords, “Vector competence” and “Insecticide resistance”, “Vector competence”, and “Insecticide exposure”. The search was not restricted to the year of publication. We selected articles that met at least two of the following characteristics to assure that the experiment design included the evaluation of the effects of IR on VC. (1) The articles were written in English; (2) the articles had to report at least two of the following parameters simultaneously: exposure to insecticides and/or evaluation of susceptibility through bioassays and/or evaluation of mechanisms of resistance to insecticides; (3) infection, dissemination, transmission, or prevalence of pathogens is reported in the results. 

All the articles were grouped according to the objective of this study; that is, the papers were organized by the level of evaluation (experiments designed for the field vs. laboratory) and by investigations that determine the influence of exposure to insecticides or resistance mechanisms on the transmission of pathogens. We omitted articles that expressed opinions, abstracts and conference proceedings, and literature that failed to describe and report forms of insecticide exposure, susceptibility testing, resistance mechanisms assays, or pathogen detection procedures. Finally, we divided articles according to the species that affect health or crops.

### 2.2. Data Extraction and Synthesis

We aimed to systematize the literature review process; all eligible articles were read, and the data were extracted in a spreadsheet. For every paper, we collected authors, year of publication, the title of the study, objectives, insect species, pathogen selected, articles studied, methods utilized for phenotypic resistance determination, kdr, and other IR-associated mutations, parameters included in life tables, metabolic resistance mechanisms (detoxifying enzymes), infection, dissemination, salivary gland, and transmission rates, susceptibility status, infection method, classification of phenotypic resistance, frequency of mutations, and the relationship between infection and resistance.

## 3. Results

The results obtained by the combination of keywords varied according to the database used. For example, a total of 1056 studies were obtained from PubMed. The analysis of the titles of published articles served to narrow the selection process. A total of 40 articles were selected for further inspection, and after reading the articles, only 30 fulfilled the selection criteria. Of these, 20 studies were associated with the influence of phenotypic IR and IR mechanisms in both field and laboratory-designed experiments; 7 studies were related to the influence of exposure to insecticides on VC. Regarding pest vectors in crops, only three studies investigated the impact of IR on VC. A complete analysis and synthesis of the articles selected are presented below.

### 3.1. Field Versus Laboratory Studies

This section compares field and laboratory research where the prevalence of infection and phenotypic or genotypic resistance are described. Most evaluations corresponding to IR and VC were carried out using insectary-reared specimens in laboratory-controlled experiments. Accordingly, the differences between field-collected organisms and those raised in controlled, optimal conditions had to be addressed.

Field research was conducted on mosquito species that transmit parasites [37,38,39,40,41,42,43,44], while only two studies involved other pathogens such as arboviruses [45,46]. Three of these studies showed a positive association between VC and IR; in other words, the presence of IR mechanisms favors VC [37,38,39]. One of them is the work of Kabula et al. [37], who analyzed susceptibility to deltamethrin in *Anopheles gambiae s. s.* mosquitoes that carried the resistant kdr allele at locus 1014 (S1014) and related it to the prevalence of *Plasmodium falciparum*. A positive association between the resistant phenotype and genotype (homozygous S1014) and the prevalence of sporozoites, the transmission stage of *P. falciparum*, was observed.

In an alternate way to study the interaction between VC and IR, infection with *P. falciparum* was enough to increase DDT mortality in field-collected kdr homozygous *An. gambiae s. s*. It is important to mention that this effect was time-dependent according to *P. falciparum* development., Increased mortality was observed one and seven days after infectious blood meals were offered (indicating ookinete development and oocyst maturation, respectively) but absent after a longer period of 14 days (sporozoite stage) [38]. Finally, Tchouakui et al. [39] demonstrated that *An. funestus* homozygous for the mutation F119 in the GST gene had higher sporozoite rates of *Plasmodium* sp.

On the other hand, results showing no association between IR and VC were reported in field-based research. For example, Collins et al. [40] discovered a non-significant association between the presence of kdr or Ace mutations and the *P. falciparum* oocyst burden in *A. gambiae s. l.* Additionally, this pattern was further confirmed since the presence of mutant alleles F1014, and Ace-1 S119 had no influence on mosquito infection with *Plasmodium* sp. in the same insect vector [41]. In *An. funestus*, the presence of a mutation in the GST gene (L119F-GST2) did not influence the prevalence of infection of *Plasmodium* sp. [39].

Besides previous studies reported in *Anopheles*, this lack of association was also reported for *Culex pipiens*, where a comparison of infection rate and oocyst burden of *P. relictum* between susceptible and field-caught resistant mosquitoes showed no significant differences between groups [42]. The only study conducted with arboviruses was reported by Anbalagan et al. [45], who demonstrated that populations of *Cx. gelidus* resistant to deltamethrin and malathion showed no infection with the Japanese encephalitis virus.

Finally, only three studies described a negative association between VC and IR. A study conducted in *Cx quinquefasciatus* and *Wuchereria bancrofti* showed a negative correlation between parasite RNA quantity (quantification of parasite burden) and esterase activity, a xenobiotic-detoxifying enzyme [43]. This negative association was also reported by Tchouakui et al. [39] in *An. funestus*; infection with *Plasmodium* sp. was more prevalent in mosquitoes lacking the mutation A296S in the GABA receptor. Regarding arboviruses, Stephenson et al. [46] analyzed the VC for DENV in populations of *Ae. aegypti* with varying kdr resistant profiles. A negative association between kdr mutations and VC was found; populations with the highest frequency of mutant alleles L1016 and C1534 had lower VC than wild-type mosquitoes (e.g., low frequency or free of kdr mutant alleles).

Studies performed in laboratory-designed experiments depict a similar picture as the results obtained in field research. Nonetheless, it must be noted that other variables such as heat treatment or varying larval density are seldomly introduced in experiments. Several studies showed no association between VC and IR, as found in field research. For example, the selection of mosquitoes with DDT showed no measurable impacts on the susceptibility of *Ae. aegypti* to dengue serotype 2 [47]. However, this investigation must be considered with caution, as details of the experimental procedure were not clearly stated. Similarly, Alto and Lord [48] demonstrated that the VC of *Ae. aegypti* raised at different larval densities and exposed to several doses of *Bacillus thuringiensis israelensis* (Bti) was not affected since no changes in infection and dissemination of dengue serotype 1 were observed. This pattern was also reported for the parasite vector *Cx. pipiens* and *P. relictum*, where a non-significant difference was found in infection rates and oocyst burden between susceptible and resistant mosquitoes with a common genetic background [42].

Contrary to these neutral results, the negative association between VC and IR was reported by Deng et al. [49], who demonstrated that after 14 days post-infection (dpi) with dengue serotype 2, deltamethrin-resistant *Aedes albopictus* showed lower values of viral concentration in the head, salivary glands, and ovaries along with lower infection rates. Additionally, it was noted that horizontal transmission to mice and vertical (progeny) transmission were diminished in resistant mosquitoes.

Regarding the presence of mechanisms of IR and their impact on VC, a similar negative pattern was found in *Anopheles funestus*. Qualitatively, *An. funestus* mosquitoes carrying a mutation in the glutathione S transferase (L119F-GSTe2) gene are prone to be mosquitoes not infected with *P. falciparum*. However, the infection intensity (as measured by oocyst count in midguts) was higher in resistant (mutation carriers) and heterozygous mosquitoes [50]. Alout et al. [51] reported these same qualitative and quantitative effects, finding that oocyst and sporozoite prevalence was greater in a strain of *An. gambiae s.s.* resistant to organophosphates and carbamates and a second strain resistant to pyrethroids and DDT. A difference in infection intensity was noted in the pyrethroid-resistant strain with a reduced oocyst burden compared to carbamate-resistant mosquitoes or the susceptible strain. No impact was observed for sporozoite burden, indicating that the transmission phase was not affected. Another study performed on *An. gambiae s. l.* showed that infection with *P. falciparum* was associated with mosquitoes carrying mutant alleles F1014 and S1014 [52].

A positive relationship between VC and IR was also reported in laboratory-designed experiments. For instance, interactions between IR and VC in *Cx. quinquefasciatus* were displayed in a pathogen-dependent manner. Mosquitoes carrying a mutation in the Ace-1 gene and duplication of an esterase were associated with higher susceptibility to infection and West Nile virus transmission but not for the Rift Valley virus [53]. Information concerning another arbovirus–vector interaction was reported by Parker-Crockett et al. [54], who demonstrated that the infection and dissemination rates of the Zika virus were superior in multi-pyrethroid-resistant populations of *Ae. aegypti* at 12 days post-infection. Moreover, resistant individuals were genotyped, and almost all individuals analyzed were characterized as homozygous resistant to I1016 and C1534 kdr mutant alleles. A similar pattern was observed for *Ae. aegypti*; permethrin-selected individuals had higher dissemination rates of dengue serotype 1 [55].

Expanding our understanding of the susceptibility of human-associated vectors to infection, aside from arboviruses or parasites, is also important due to the use of fungal entomopathogens to control vector populations. The two entomopathogens, *Metarhizium anisopliae* and *Beauveria bassiana*, displayed a similar tendency regarding IR and susceptibility to infection. Mosquitoes *An. gambiae s. s.*, selected with pyrethroids and carriers of a kdr mutation (L1014F), were more susceptible to infection by these fungi than wild-type mosquitoes [56].

Only a few investigations describe the correspondence between field and laboratory studies. In particular, Saddler et al. [57] reported a laboratory-reared DDT-resistant colony of *An. gambiae* showed an increase in DDT sensitivity after challenge with *P. berghei*, similar to the findings of Alout et al. [38] in field-collected *An. gambiae s. s.* and *P. falciparum*. Field results were also confirmed in the laboratory by McCarroll and Hemingway [43]; laboratory findings reassured that high esterase activities were negatively correlated with *W. bancrofti* prevalence to such extent as to completely inhibit parasite infection. Finally, Vézilier et al. [42] found the same non-significant pattern of prevalence and intensity of infection in *Cx. pipiens* infected with *P. relictum* in either laboratory-designed or field experiments.

The first objective of this systematic review is to compare the results of the relationship between IR and VC in field-collected insects versus insects reared in optimal, controlled environments. Before drawing any conclusion, we must delimit the compendium of studies involved. Field research is scarcer, and, at the time of writing this manuscript, only nine published articles were found, four of which were conducted in *An. gambiae* in association with *P. falciparum*. Interestingly, there were only two studies with arboviruses vectors (Table 1).

It was not possible to find one single clear trend to determine the direction and magnitude at which IR affects VC. Three patterns were found: IR positively affects VC, thus enhancing VC [37,38,39]; IR negatively affects VC, thus diminishing VC [39,43,46]; and IR has no influence on VC [39,40,41,42,45]. More studies are needed to determine what factors dictate the observed patterns. However, it might be hypothesized that these trends are species-specific since it was found that IR positively affected VC or infection susceptibility in *An. gambiae s. s.* [37,38,51,56] but not in *An. gambiae s. l.* [40,41], and, in the case of *Cx. quinquefasciatus*, IR negatively impacted VC [43]. Again, more research must be done to confirm the hypothesis that the effect of IR depends on the insect species. Another important consideration of field research is the physiological status of field-collected larvae. For example, poor larval nutrition reduces VC in *An. gambiae s. s.* [58], and nowadays, it is well known that microbiota shapes the VC of vectors of human disease [59]. IR mechanisms could be another way to explain the differential impacts of IR on VC, as the negative association found in *Cx. quinquefasciatus* was related to the increased activity of an esterase, a metabolic IR mechanism [43]. By contrast, target site modifications (kdr mutations) found in *An. gambiae* were present in the studies where no impacts or positive associations were described [37,38,40,41,51,52].

Results from insects raised in controlled environments show the same three patterns of the influence of IR on VC observed in field research, although studies are also scarce. Furthermore, investigations in laboratory experiments involved arbovirus vectors, and only three articles described the results from vectors of *P. falciparum* [50,51,52]. In the case of neutral results [42,47,48,53], two works focused on exposure to insecticides without IR mechanisms being analyzed [47,48], and the other only examined the effect of IR mechanisms on VC [43,50,51,52,53,54,56,57]. Only one study [55] evaluated phenotypic resistance and IR mechanisms (Table 1).

The hypothesis that IR influences VC in a species-specific manner, as observed in results from fieldwork, is not supported in the studies conducted in the laboratory. In other words, results obtained from laboratory experiments do not depend on the insect species evaluated. Even though the proposed hypothesis is rejected, a cautious interpretation is needed since most works were performed with arbovirus vectors instead of parasite vectors in field studies.

Additionally, it is important to mention that rearing insects under controlled, optimal conditions might broaden differences with insects found in field environments in which traits such as longevity, blood-feeding behavior, and infection determinants can vary vastly [60]. For example, it has been determined that laboratory rearing increases the fitness of some groups of insects (e.g., Diptera and Hymenoptera) because of the relaxation of selection pressures [61]. This phenomenon could impact some biological characteristics; for instance, Shi et al. [62] reported a lower virome diversity in laboratory-reared *Ae. albopictus* when compared to field-collected mosquitoes. This same pattern was found for bacteria; a qualitative difference in midgut microbiota has been seen between laboratory-reared and field-collected mosquitoes such as *Ae. aegypti*, *Ae. albopictus*, and *Cx. quinquefasciatus* [63]. Regarding the immune response, the reduction of microbial exposure and the high degree of inbreeding of laboratory-reared mosquitoes could impose a cost in genetic variability that can affect the repertoire of molecules used to recognize pathogens and, thus, increase the differences in VC between field and laboratory, as is reported in *Anopheles* [64]. Differential regulation of immune elements such as phenoloxidase activity in other vector species has been shown as reported for *Cx. pipiens* raised in optimal conditions within an insectary versus field-caught mosquitoes [65]. More research is needed to determine the influence of environmental factors and their impact on VC.

There are multiple variables in experimental designs, even in laboratory studies, that can affect the interpretation of results. For example, there should be an agreement on how infectious bloodmeals are offered to insects, what strategies of pathogen identification are utilized, what IR mechanisms are evaluated, what methods are used to determine phenotypic IR, and under what criteria phenotypic IR is established. This agreement could enhance the interpretation of results and help us determine if current vector control strategies should be used despite the presence of IR. We can also highlight the importance of establishing reproducible experiments to assess the impact of IR on VC.

### 3.2. Effects of Insecticide Exposure on Pathogen Transmission

One key aspect that must be considered while exploring current control efforts’ effectiveness is determining if insecticide exposure could impair or enhance VC. It is possible that exposure to sub-lethal doses of insecticides could decrease insects’ capacity to acquire a pathogen, limiting infection capacity. As this factor remains to be elucidated completely, a contrary enhancing pattern may be present, as reported by Muturi and Alto [66], Moltini-Conclois et al. [67], and Knecht et al. [68]. In this section, we show evidence supporting either of the two scenarios.

Regarding enhancement of VC after exposure to insecticides, Muturi and Alto [65] found increased viral infection and dissemination of the Sindbis virus in *Ae. aegypti* when larvae were exposed to malathion. However, heat treatment at 30 °C was also applied to immature stages; results must be interpreted with caution as temperature may influence VC [69]. The same effect was found in *Ae. aegypti* exposed to Bti. Here, the larval exposure augmented dengue infection and dissemination in two Bti-resistant strains of *Ae. aegypti* [67].

Insecticide susceptibility could interact with physiological characteristics such as insect age. For example, older *Ae. albopictus* mosquitoes exposed to sublethal doses of bifenthrin develop higher dissemination viral titers of Zika virus than unexposed old (11–12 days old) and younger mosquitoes (6–7 days old). Remarkably, older mosquitoes exposed to bifenthrin exhibited greater viral dissemination to other tissues outside the midgut, even when compared with younger exposed mosquitoes [68].

On the other hand, studies that show an impairment of VC after insecticide exposure are scarcer. Oral consumption of bifenthrin has been shown to reduce dengue infection rates and body titers (dissemination) at 14 dpi in *Ae. albopictus*, but no effect was observed at 7 dpi [70]. The same trend was consistently found in *An. gambiae s. s.* collected in Uganda. Homozygous mosquitoes for the kdr mutation L1014S had a reduction in prevalence and infection intensity by *P. falciparum* after exposure to deltamethrin in contrast to the unexposed control group [44]. Finally, Hauser et al. [71] determined that exposing insects at the larval or adult stage or both stages to permethrin diminished VC of *An. gambiae s. s.* for *P. berghei*.

As reported in the previous section, a neutral trend is found in the relationship between insecticide exposure and VC. Alomar et al. [72] reported that exposure to pyriproxyfen had no impact on the infection, dissemination, or transmission rates of the Zika virus in *Ae. aegypti*.

In this section, we have aimed to review all information regarding the exposure of insects to pesticides; nonetheless, as in the previous section, few heterogeneous studies are published. At the time of writing this review, only seven studies strictly adhered to the direct exposure of immature stages or adult insects to any pesticide and the further evaluation of any component of VC. Besides the small number of studies, there is variation in experimental settings that limits our ability to establish clear conclusions about the influence of insecticide exposure on VC. For example, only one study was performed in the *Anopheles*-*Plasmodium* association [44]; the remaining were conducted in *Ae. aegypti* or *Ae. albopictus* [66,67,68,70,72]. To expand this variation, insecticides used for bioassays were from different toxicological groups possessing different modes of action (malathion, an organophosphate [66]; Bti, a biological insecticide [67]; pyriproxyfen, a juvenile hormone analog [72]; bifenthrin [68,70]; permethrin [71]; and deltamethrin [44]). Three studies focused on insecticide exposure in larvae [65,66,72], while the other four were performed in adults [67,69,70,71]; finally, there is also variation in the pathogen used for infections; two studies involved the dengue virus [66,69] (Table 1).

We can synthesize most of the studies into two patterns: one shows that exposure to insecticides enhances VC [65,66,67]; on the other hand, a contrary, negative effect could also be observed [69,70,71]. Only one study showed a third pattern in which insecticide exposure had no influence on VC [72]. Two covariates, mosquito age, and heat treatment, are present in the positive studies, complicating the interpretation of results regarding the direct effects of insecticide exposure on VC. These covariates are reported to influence VC; Pigeault et al. [73] observed that older *Cx. pipiens* had less prevalence and intensity of infection of *P. relictum* than younger mosquitoes. This contrasts with the results of Knecht et al. [68], where older exposed mosquitoes exhibited higher dissemination rates of the Zika virus. This means that insecticide exposure could be a factor that promotes changes in VC but only in combination with other biological characteristics such as insect age. As for the previous section, there is a need for more studies with standardized experimental designs on the relationship between IR and VC to assure comparable results.

### 3.3. Impacts of Insecticide Resistance on Pest Vectors in Crops

It has been noted that patterns found in vectors of human pathogens are also present in crop pests. To illustrate this relationship, Wan et al. [74] found that spinosad-resistant *Frankliniella occidentalis* had higher viral replication, dissemination, and transmission of *tomato spotted wilt orthotospovirus* (TSWV), contrasting with its susceptible counterpart. These results were obtained only in scenarios where short acquisition periods of the pathogen were evaluated. Regarding life parameters, it is remarkable that resistant *F. occidentalis* showed a prolonged pre-adult stage, a reduced life span, and lower pupation and sex ratios than its susceptible counterpart. Concerning *F. occidentalis* IR mechanisms, it has been pointed out that several of these mechanisms resemble those found in human-associated vectors, including decreased penetration of insecticides, detoxifying enzymes, and target resistance. Zhang et al. [75] reported evidence of these similarities, who described genes associated with insecticide resistance using metagenomic approaches and alteration of immune pathways in TSWV-infected *F. occidentalis*.

Another alteration in VC is observed in *Myzus persicae*, the peach potato aphid. Here, pyrethroid-susceptible individuals (Type J) displayed less acquisition of potato virus Y in λ-cyhalothrin sprayed leaves than the control (non-sprayed) leaves even three days after spraying. When repeating this experiment using a resistant strain (Type O) characterized by an Ace and a kdr mutation in M918L, the spraying of leaves did not produce a reduction in acquisition of potato virus Y by *M. persicae* type O. It can be hypothesized that the presence of IR mechanisms affects viral acquisition [76]. In contrast to these findings, no association was determined between IR and VC. Zhao et al. [77] found that, after 48 h, there was no difference in transmission rates between susceptible and spinosad-resistant *F. occidentalis* individuals. This difference could arise given that IR alters only acquisition events and not the molecular machinery related to the transmission of pathogens [74].

All the studies mentioned earlier were conducted in laboratory settings, and less is known regarding how field variants (e.g., different biotypes) could affect this relationship. Studies conducted in whiteflies (*Bemisia tabaci*) have reported variations in IR as well as genes related to the transmission of *tomato yellow leaf curl virus* (TYLCV) and *tomato crinivirus* (TC) among Middle East-Asia Minor 1 (MEAM 1) and Asia II 1 species. It is important to specify the variation of genes involved in IR, such as acetylcholinesterase like protein and cytochrome P450, proteins acting as target sites for carbamates/organophosphates and detoxifying enzymes for pyrethroids, respectively [78]. On the other hand, protein variants involved in virus transmission were also detected for TYLCV, such as an aldo-ketoreductase and elicitin-like protein 6, in addition to variants for viral transmission of tomato crinivirus such as 70 Da heat shock protein, AAA-ATPase-like domain-containing protein, alpha-glucosidase, and others [78]. Given this example, it is essential to evaluate how this could enhance or hamper VC in the light of IR. Considering these small number of research advances, we can look at fine-scale variation in the relationship between VC and IR in non-human associated vectors. First, it is apparent that, in controlled situations, IR alters the VC of two vectors of crops [74,76,77]. Second, this alteration is complex and remains to be investigated and expanded to other insect species as well as factors such as immune system interplay and evaluation of individuals exposed to insecticides but with one or several resistance mechanisms (Table 1).

**Table 1:** Studies aimed at the relationship between insecticide resistance (IR) or exposure to insecticides and vector competence (VC).

Species	Pathogen	Insecticide Exposure	Metabolic Resistance	Target Site Modifications	Phenotypic Resistance	Type of Association	Location	Additional Treatments	Reference
*Anopheles gambiae*	*Plasmodium berghei*	DDT	GST		DDT	Positive	Lab		[57]
*An.gambiae s.s.*	*Plasmodium falciparum*			L1014S	Deltamethrin	Positive	Field		[37]
	*Plasmodium falciparum*	DDT		L1014F	DDT	Positive	Field		[38]
	*Plasmodium falciparum*			L1014F, G119S	OP, CAR, and PYR-DDT	Negative ^1^	Lab		[51]
	*Plasmodium falciparum*			L1014F, G119S	OP, CAR, and PYR-DDT	Positive ^2^	Lab		[51]
	*Metarhizium ^3^ anisopliae*			L1014F	PYR	Positive	Lab		[56]
	*Beauveria bassiana ^3^*			L1014F	PYR	Positive	Lab		[56]
	*Plasmodium falciparum*	Deltamethrin		L1014S		Negative	Field		[44]
	*Plasmodium berghei*	Permethrin				Negative	Lab	Larval competition	[71]
*An.s gambiae s. l.*	*Plasmodium falciparum*	α-Cypermethrin, Deltamethrin, Permethrin		N1575Y, I1527T, L1014F, G119S	PYR	Neutral	Field		[40]
	*Plasmodium* sp.			L1014F, G119S		Neutral	Field		[41]
	*Plasmodium falciparum*			L1014F, L1014S		Positive	Lab		[52]
*An. funestus*	*Plasmodium falciparum*			L119F-GSTe2		Negative ^1^	Lab		[50]
	*Plasmodium falciparum*			L119F-GSTe2		Positive ^2^	Lab		[50]
	*Plasmodium* sp.			L119F-GSTe2		Neutral	Field		[39]
	*Plasmodium* sp.			L119F-GSTe2		Positive	Field		[39]
	*Plasmodium* sp.			A296S (GABA)		Negative	Field		[39]
*Culex gelidus*	*Japanese Encephalitis Virus*				Deltamethrin, Malathion	Neutral	Field		[45]
*Cx. pipiens*	*Plasmodium relictum*			Ester, AceR		Neutral	Field		[42]
	*Plasmodium relictum*			Ester, AceR		Neutral	Lab		[42]
*Cx. quinquefasciatus*	*Wuchereria bancrofti*		Esterase activity			Negative	Field		[43]
	*Wuchereria bancrofti*		Esterase activity			Negative	Lab		[43]
	WNV			G119S, Ester	OP	Positive	Lab		[53]
	RVV			G119S, Ester	OP	Neutral	Lab		[53]
*Aedes aegypti*	DENV-2	DDT				Neutral	Lab	Heat shock	[47]
	DENV-1	Bti				Neutral	Lab	Larval densities	[48]
	Zika			V1016I, F1534C	PYR	Positive	Lab		[54]
	Sindbis	Malathion				Positive	Lab	Heat treatment	[66]
	DENV	Bti			Bti	Positive	Lab		[67]
	ZIKV	Pyriproxyfen				Neutral	Lab		[72]
	DENV-1		CYP and GST	V1016I, F1534C	PYR	Positive	Lab		[55]
	DENV			V1016I, F1534C		Negative	Field		[46]
*Ae. albopictus*	DENV-2				Deltamethrin	Negative	Lab		[49]
	Zika	Bifenthrin				Positive	Lab		[68]
	DENV	Bifenthrin				Negative	Lab		[70]
*Frankliniella occidentalis*	Tomato spotted wilt orthotospovirus				Spinosad	Positive	Lab		[74]
	Tomato spotted wilt orthotospovirus				Spinosad	Neutral	Lab		[77]
*Myzus persicae*	Potato Virus Y	λ-Cyhalothrin		Ace, M918L	Diethyl carbamates PYR	Positive	Lab		[76]

OP, organophosphates; CAR, carbamates; PYR, pyrethroids. ^1^ Indicates that the negative influence of IR on VC was detected in the prevalence of infection. ^2^ Indicates that the positive impact of IR or exposure to insecticides was detected in the pathogen’s burden (intensity of infection. ^3^ They were included to show the relationship between infection susceptibility and IR; these are independent of VC.

## 4. Conclusions

There is currently a need to determine the consequences that IR can have on vector control programs and, in turn, on the transmission of pathogens. In this review, we aimed to establish the influence of IR and exposure to insecticides on the ability of organisms to become infected, maintain infection, and transmit a pathogen, that is, their VC. Although current information is scarce and obtained under highly heterogeneous experimental designs, evidence indicates that insecticide exposure and resistance increase the risk of pathogen transmission. Under this context, adverse effects such as epidemics in human populations or economic repercussions on crops will increase while current vector control efforts become entirely ineffective. However, studies also point to opposite effects where IR or exposure to insecticides reduces VC. To determine the impact of IR on VC precisely, it is essential to establish reproducible experimental designs to reduce the presence of confusing variables that make the interpretation of results difficult. Therefore, although there is evidence related to the influence of IR on VC, more research is necessary.

## Data Availability

Not applicable.
